# Clinical pathway data engineering framework (CPDEF): a reproducible methodology for constructing machine learning-ready case-mix datasets from hospital information systems

**DOI:** 10.1016/j.mex.2026.104163

**Published:** 2026-09-13

**Authors:** Suryanto Nugroho, Raden Venantius Hari Ginardi, I Ketut Eddy Purnama

**Affiliations:** a Doctoral Program in Technology Management, Interdisciplinary School of Management and Technology , Institut Teknologi Sepuluh Nopember, Surabaya, Indonesia; b Department of Information Technology, Faculty of Intelligent Electrical and Informatics Technology, Institut Teknologi Sepuluh Nopember, Surabaya, Indonesia; c Department of Computer Engineering, Faculty of Intelligent Electrical and Informatics Technology, Institut Teknologi Sepuluh Nopember, Surabaya, Indonesia; d Department of Informatics, Universitas Muhammadiyah PKU Surakarta, Surakarta, Indonesia

**Keywords:** Keywords: clinical pathway, Data engineering, Hospital information systems, Case-mix classification, INA-CBG, Machine learning readiness, Information management

## Abstract

Healthcare analytics, clinical pathway analysis, predictive modeling, and artificial intelligence applications require large-scale and well-structured datasets derived from routine hospital operations. However, hospital information systems often store diagnoses, procedures, reimbursement classifications, and hospitalization outcomes in heterogeneous formats that are difficult to integrate into analytical workflows. This article presents the Clinical Pathway Data Engineering Framework (CPDEF), a reproducible healthcare data engineering methodology for constructing machine learning-ready case-mix datasets from hospital information systems.

CPDEF integrates ICD-10 diagnoses, ICD-9-CM procedures, INA-CBG classifications, and length-of-stay information into a standardized analytical repository. The framework was applied to 165,391 inpatient hospitalization episodes collected between November 2016 and April 2026 and produced a repository containing 32 standardized variables suitable for healthcare analytics and artificial intelligence applications. Validation demonstrated deterministic workflow execution, structural consistency, coding-system integrity, and compatibility with standard machine learning workflows, supporting the reproducibility of the proposed methodology. CPDEF provides a transparent and scalable workflow for transforming heterogeneous hospital data into machine learning-ready analytical resources.

## Highlights

• Provides a reproducible healthcare data engineering framework for constructing standardized case-mix datasets from hospital information systems.
• Integrates diagnoses, procedures, reimbursement classifications, and hospitalization outcomes into a unified analytical repository.
• Supports healthcare analytics, clinical pathway analysis, predictive modeling, and artificial intelligence applications.

## Graphical abstract

**Figure:**
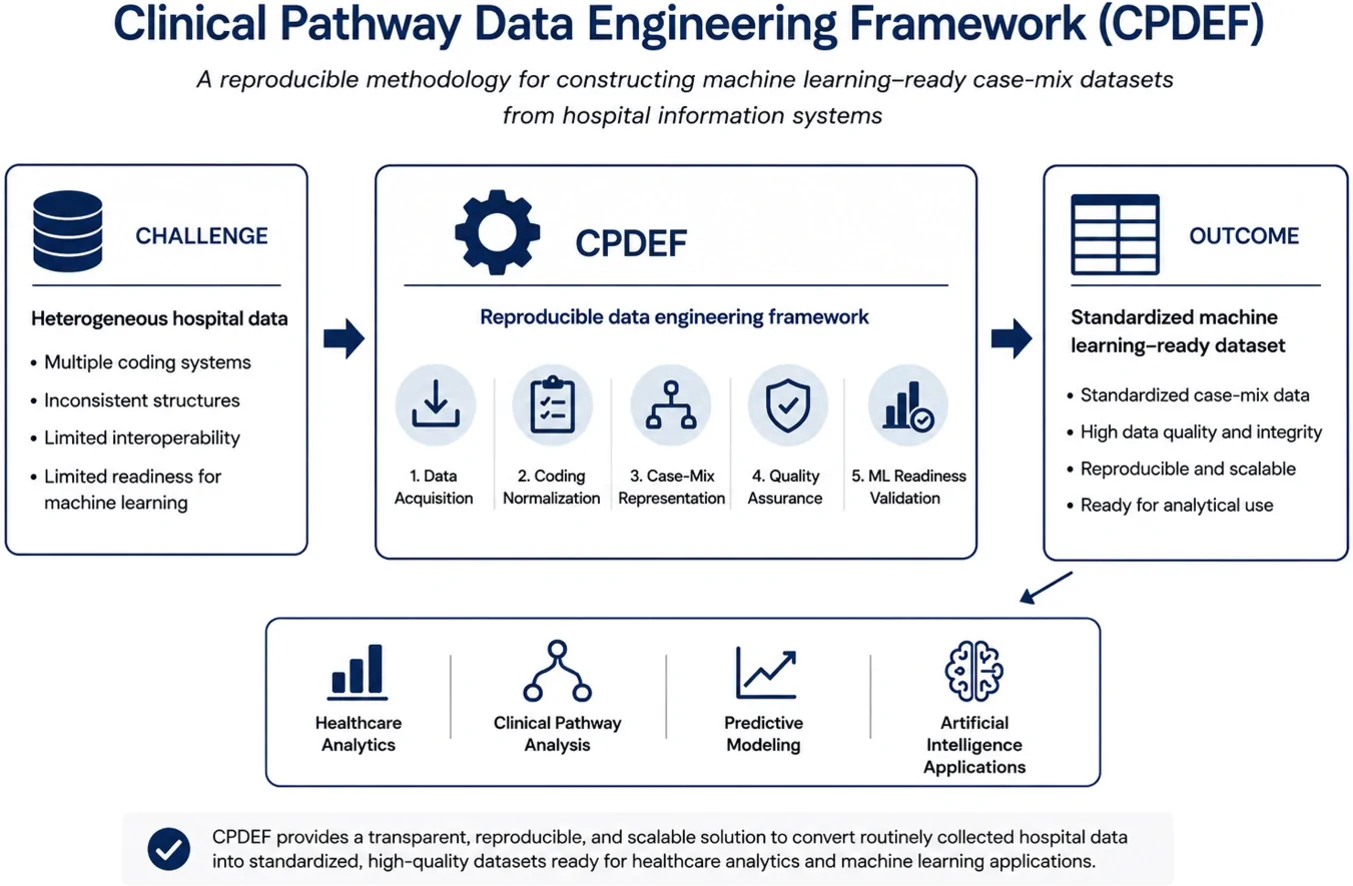

Specifications table.

**Table:** 

**Subject area**	Computer Science
**More specific subject area**	Clinical pathway analytics, healthcare data engineering, and machine learning-ready healthcare datasets
**Name of your method**	Clinical Pathway Data Engineering Framework
**Name and reference of original method**	Not applicable
**Resource availability**	The curated dataset used in this study is publicly available at Zenodo (https://doi.org/10.5281/zenodo.20588385). The complete CPDEF software implementation supporting the methodology is also openly available at Zenodo (https://doi.org/10.5281/zenodo.21817222). Together, these resources enable full methodological reproducibility without the need for patient-identifiable information.

## Background

The widespread adoption of EHRs, HIS, and diagnosis-related group reimbursement mechanisms has resulted in the generation of substantial volumes of healthcare data during routine clinical operations [1]. These data contain valuable information regarding patient diagnoses, medical procedures, treatment pathways, healthcare resource utilization, hospitalization outcomes, and reimbursement classifications [2]. Such information provides opportunities for healthcare analytics, clinical pathway evaluation, hospital performance monitoring, process mining, digital twin development, and machine learning applications.

Despite the availability of large healthcare databases, transforming routine operational healthcare records into standardized machine learning-ready analytical repositories remains a significant challenge. Healthcare data are frequently distributed across multiple information systems, stored in heterogeneous formats, and organized according to operational rather than analytical requirements [3]. Diagnosis information, procedure information, reimbursement classifications, and hospitalization outcomes are often represented in separate database structures, thereby requiring extensive and often institution-specific preprocessing before analytical use [4].

Standardized coding systems have been widely adopted to improve interoperability, reimbursement, and secondary use of healthcare data. Diagnosis information is commonly represented using the International Classification of Diseases, Tenth Revision (ICD-10), whereas procedural interventions are documented using the ICD-9-CM. Reimbursement and case-mix classifications are frequently represented through DRG systems or equivalent national case-mix frameworks. The INA-CBG system serves as the national reimbursement mechanism and categorizes hospitalization episodes according to diagnoses, procedures, severity levels, and expected healthcare resource utilization [5]. These coding systems standardize clinical representation but do not specify how heterogeneous operational records should be transformed into reproducible analytical repositories.

Although these coding systems provide standardized clinical and administrative representations, they do not define reproducible protocols for transforming heterogeneous operational hospital databases into standardized analytical repositories suitable for downstream machine learning and healthcare analytics. Existing studies predominantly emphasize predictive modeling, clinical outcome prediction, or healthcare optimization, whereas the upstream data engineering procedures required to construct analytical repositories are rarely described in sufficient detail for independent implementation. Researchers frequently develop institution-specific preprocessing workflows across healthcare organizations that are difficult to reproduce, compare, or adapt [6] (Table 1,2,3,4,5,6, 7,8,9,10).

**Table 1:** Workflow of machine learning-oriented case-mix representation.

Step	Operation	Repository Output
1	Read the finalized INA-CBG reimbursement code	Raw administrative code
2	Verify the code against the official INA-CBG reference	Verified INA-CBG code
3	Retrieve the official description	Standardized reimbursement description
4	Store the reimbursement code	INACBG
5	Store the standardized description	DESCRIPTION
6	Integrate with ICD-10 diagnoses, ICD-9-CM procedures, and LOS	standardized analytical repository

**Table 2:** Fixed slot encoding rules used in the CPDEF.

Repository Component	Encoding Rule
ICD-10 diagnosis codes	Preservation of original ICD-10 codes without modification
ICD-9-CM procedure codes	Preservation of original ICD-9-CM codes without modification
Primary Diagnosis	Assigned to Diagnosis_1
Secondary Diagnoses	Sequentially assigned to Diagnosis_2–Diagnosis_12
Primary Procedure	Assigned to Procedure_1
Secondary Procedures	Sequentially assigned to Procedure_2–Procedure_17
Missing Values	Retained as missing structural values
Missing Value Imputation	Not performed
Maximum diagnostic variables	12
Maximum procedure variables	17

**Table 3:** Data quality assurance procedures (DQA procedures).

Quality Component	Verification Procedure
Missing Values	Assessment of structural completeness
Duplicate Records	Duplicate verification of episodes
ICD-10 Codes	Verification of format and coding
ICD-9-CM Codes	Verification of format and coding
INA–CBG Codes	Verification of structural consistency
LOS Values	Verification of logical consistency

**Table 4:** Repository components.

Component	Description
Analytical Dataset	Standardization of the hospitalization records
Variable Dictionary	Definitions and Variable Descriptions
Metadata Documentation	Characteristics and Structure of the Dataset
Validation Documentation	Quality assurance records
Workflow Documentation	Data engineering procedures

**Table 5:** Software environment.

Component	Software
Programming Language	Python 3.10.20
Data Processing	Pandas 2.2.3
Numerical Computation	NumPy 2.3.5
Machine learning utilities	Scikit-learn 1.7.1
Statistical Analysis	SciPy 1.16.1
Visualization	Matplotlib 3.10.5
Development Environment	Jupyter Notebook version 6.0.3

**Table 6:** Structural validation results.

Indicator	Value
Hospitalization Episodes	165,391
Total Variables	32
Diagnosis Variables	12
Procedure Variables	17
Case-Mix Variables	2
Outcome Variables	1
Repository Status	Successfully generated

**Table 7:** Validation of clinical coding.

Coding System	Validation Procedure	Status
ICD-10	Format verification	Passed
ICD-9-CM	Format verification	Passed
INA–CBG	Structural verification	Passed
LOS	Verification of the logical consistency	Passed

**Table 8:** Reproducibility assessment.

Execution Run	Records Generated	Variables Generated	Output
Run 1	165,391	32	Identical
Run 2	165,391	32	Identical
Run 3	165,391	32	Identical

**Table 9:** Machine learning readiness indicators.

Indicator	Status
Construction of the feature matrix	Successful
Categorical Encoding	Successful
Classification Compatibility	Successful
Regression Compatibility	Successful
Analytical Reusability	Successful

**Table 10:** CPDEF positioning relative to existing healthcare data frameworks.

Framework	Primary Objective	Primary Output	ML-Ready Repository	Native INA-CBG Support
OMOP CDM[7]	Semantic interoperability	Common data model	Partial	No
HL7 FHIR[8]	Exchange of healthcare information	standardized interoperable resources	No	No
MIMIC-IV[9]	Public Clinical Research Database	Curated research dataset	Yes	No
DRG Systems[10]	Case-mix reimbursement	Reimbursement classification	No	No
CPDEF	Reproducible healthcare data engineering	Standardized ML-ready administrative repository	Yes	Yes

To address these limitations, this study proposes the Clinical Pathway Data Engineering Framework (CPDEF), a reproducible methodology for constructing standardized machine learning-ready case-mix repositories directly from routine hospital information systems. CPDEF integrates five sequential stages within a unified and reproducible workflow, including case-mix data acquisition and episode construction, clinical coding normalization, machine learning-oriented case-mix representation, data quality assurance, and machine learning readiness validation. The methodology was implemented using 165,391 inpatient hospitalization episodes containing ICD-10 diagnoses, ICD-9-CM procedures, INA-CBG classifications, and length-of-stay information collected between November 2016 and April 2026.

The primary contribution of CPDEF is the documentation of a reproducible data engineering protocol for transforming heterogeneous hospital administrative data into standardized analytical repositories rather than the development of a predictive model. CPDEF improves methodological transparency, facilitates reproducibility, and provides a reusable foundation for healthcare analytics, process mining, machine learning, digital twin development, and hospital operations research by explicitly documenting every transformation stage, repository structure, validation procedure, and implementation protocol.

## Method details

### Clinical pathway data engineering framework

The Clinical Pathway Data Engineering Framework (CPDEF) was developed as a reproducible healthcare data engineering methodology for constructing machine learning-ready case-mix datasets from routine hospital information systems. The framework addresses the challenges associated with integrating heterogeneous clinical coding systems, reimbursement classifications, and hospitalization outcomes into a standardized analytical repository suitable for secondary data analysis.

CPDEF introduces a unified workflow that combines clinical coding normalization, case-mix representation based on machine learning, data quality assurance, and analytical readiness validation, unlike conventional data preparation approaches that primarily focus on data extraction and cleaning. The methodology transforms routinely collected hospitalization records into a reusable dataset that supports healthcare analytics, clinical pathway analysis, predictive modeling, and artificial intelligence applications.

The framework comprises five sequential stages:

1) Case-Mix Data Acquisition.
2) Clinical Coding Normalization.
3) Machine Learning-Oriented Case-Mix Representation.
4) Data quality assurance.
5) Validation of machine learning readiness.

**Fig. 1:**
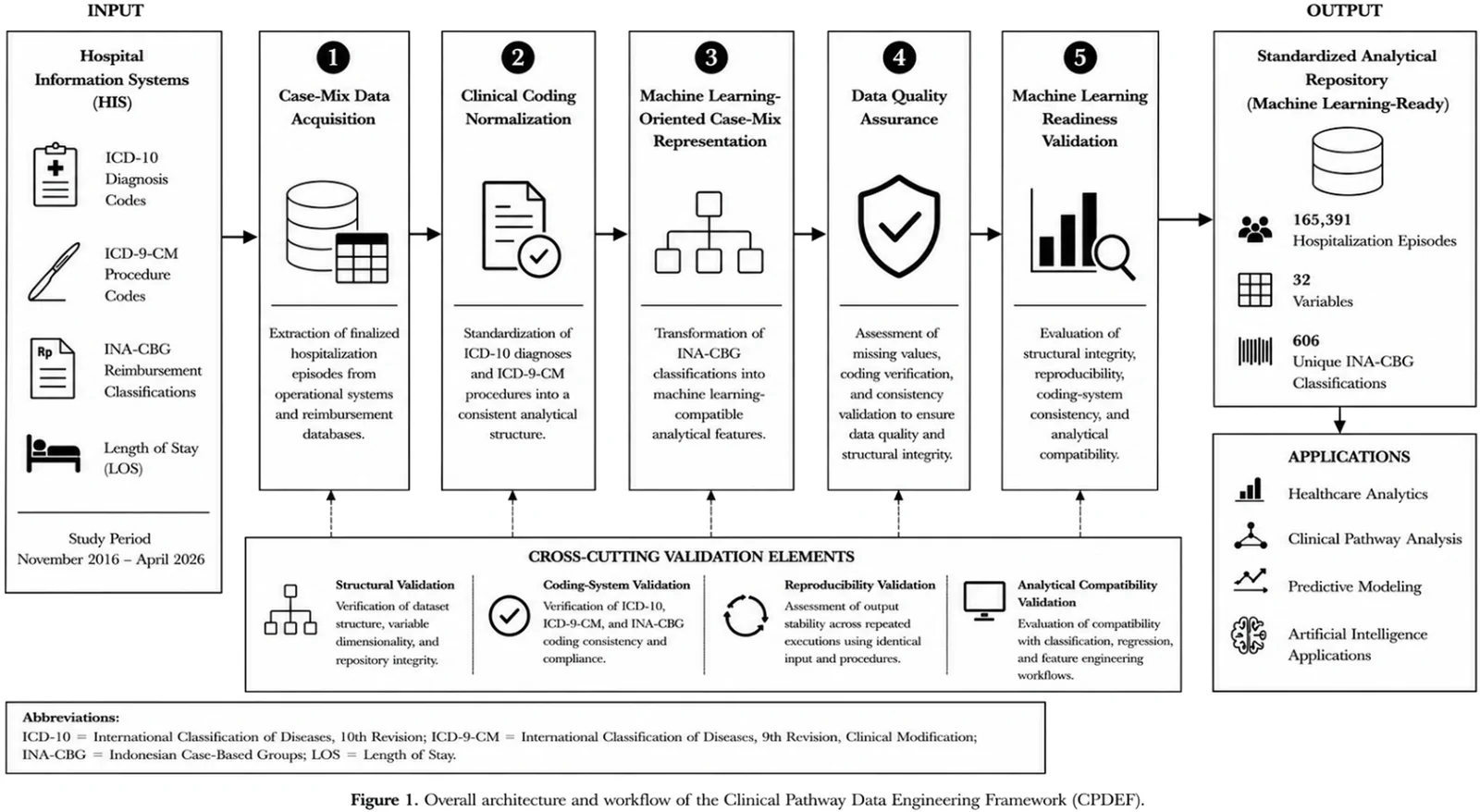
Overall workflow of the clinical pathway data engineering framework.

CPDEF integrates ICD-10 diagnosis codes, ICD-9-CM procedure codes, INA–CBG reimbursement classifications, and LOS information into a standardized analytical structure. The framework was applied to 165,391 inpatient hospitalization episodes collected between November 2016 and April 2026, and a machine learning-ready repository containing 32 analytical variables was generated. Fig. 1 shows the overall architecture and workflow of the proposed methodology.

The framework was implemented using five relational modules extracted from the hospital information system: admission, medical record, diagnosis, procedure, and billing modules. To integrate diagnoses, procedures, reimbursement classifications, and hospitalization outcomes into a single analytical record, individual hospitalization episodes were reconstructed using a unique episode identifier. Only the finalized inpatient episodes were included in the repository. All direct patient identifiers were removed through a structured de-identification process before repository construction to ensure compliance with institutional ethical requirements. The reconstructed hospitalization episodes were the standardized input for all subsequent stages of the CPDEF workflow.

### Case-Mix data acquisition

The first stage of CPDEF focuses on acquiring and reconstructing hospitalization episodes from the hospital information system. The source database consisted of five relational operational modules: admission, medical record, diagnosis, procedure, and billing. The admission module contained hospitalization episode identifiers and admission and discharge information. The medical record module contained episode-level administrative information. Diagnosis records were maintained using ICD-10 codes, procedural interventions were recorded using ICD-9-CM codes, and finalized INA-CBG reimbursement classifications were obtained from the Billing module after the reimbursement process was completed.

Data integration was performed using the unique hospitalization episode identifier as the primary relational key. Only finalized inpatient hospitalization episodes were included in the repository construction. Prior to data integration, all direct patient identifiers, including patient names, medical record numbers, national identification numbers, addresses, telephone numbers, and other PII, were removed through a structured de-identification procedure. Each hospitalization episode was represented only by an internal episode identifier used exclusively during repository construction. No identifiable patient information was retained in the published analytical repository.

Finalized INA-CBG classifications were extracted directly from the hospital billing module after the reimbursement process was completed. Throughout the study period (November 2016 to April 2026), hospitalization episodes retained the finalized INA-CBG classifications assigned according to the official INA-CBG tariff version implemented by the hospital at the time of reimbursement. When national tariff updates occurred, historical records were not regrouped or recorded retrospectively. Consequently, the CPDEF preserves the original reimbursement classifications recorded in the hospital information system while maintaining consistency with the corresponding administrative and clinical documentation.

### Machine learning-oriented case-mix representation

Traditional healthcare reimbursement classifications are primarily designed to support administrative, financial, and resource allocation processes rather than ML applications. The direct use of case-mix classifications as analytical variables may introduce challenges related to high-cardinality categorical features, sparse representations, and limited interpretability within predictive modeling workflows [11].

Machine learning-oriented case-mix representation within CPDEF does not generate additional reimbursement-derived variables. Instead, the framework standardizes finalized INA-CBG classifications by preserving both the official reimbursement code and its corresponding clinical description within the analytical repository. This approach preserves clinically meaningful reimbursement information while maintaining compatibility with downstream analytical applications.

The representation strategy preserves the reimbursement classification’s original semantic meaning while ensuring consistent integration with diagnosis codes, procedure codes, and hospitalization outcomes. The framework enables reimbursement information to be incorporated into downstream analytical workflows without altering the original case-mix classification by maintaining standardized administrative variables within the analytical repository [12].

The finalized INA-CBG code was extracted from the billing module for each hospitalization episode after the completion of the reimbursement process. Subsequently, the extracted code was verified against the official INA-CBG reference to ensure structural consistency before assigning its corresponding standardized description. Invalid or unmatched reimbursement codes were flagged for further review during the quality assurance stage. Therefore, the repository contains two administrative variables: INACBG (official reimbursement code) and DESCRIPTION (official INA-CBG clinical description).

For example, a finalized reimbursement record containing the INA-CBG code C-4–13-II is represented in the repository as INACBG = C-4–13-II, while its corresponding official reimbursement description is stored as DESCRIPTION = Chemotherapy (Moderate). This representation enables the consistent integration of reimbursement information with ICD-10 diagnoses, ICD-9-CM procedures, and hospitalization outcomes within the same hospitalization episode without modifying the original reimbursement classification. Consequently, reimbursement information remains fully traceable to the original hospital information system while becoming directly available for machine learning, healthcare analytics, clinical pathway analysis, and secondary data reuse.

Pseudocode of ML–oriented case-mix representation.

**Table:** 

Algorithm 1. Machine Learning-Oriented Case-Mix Representation

Input:
Finalized INA-CBG reimbursement records

Process:
1. Finalized INA-CBG codes are extracted from the billing module.
2. Each code is verified against the official INA-CBG reference.
3. The corresponding standardized reimbursement description is assigned.
4. The reimbursement code is stored as the INACBG variable.
5. The standardized description is stored as the DESCRIPTION variable.
6. Integrate both variables with ICD-10 diagnoses, ICD-9-CM procedures, and LOS.

Output:
Standardized case-mix representation (CMR)

### Feature integration and construction of the analytical data set

CPDEF integrates standardized diagnostic variables, procedure variables, administrative case-mix variables, and hospitalization outcomes into a unified analytical repository. Variables from the admission, diagnosis, procedure, medical record, and billing modules were linked using a unique hospitalization episode identifier, enabling reconstruction of each finalized inpatient encounter as a single episode-level analytical record.

Hospital information systems typically store diagnoses, procedures, reimbursement classifications, and outcomes across separate operational modules. CPDEF consolidates these data into a standardized tabular structure containing ICD-10 diagnosis codes, ICD-9-CM procedure codes, INA-CBG classifications, corresponding reimbursement descriptions, and length-of-stay information [13]. This structure preserves the original coding and reimbursement information while supporting downstream healthcare analytics, machine learning, and validation procedures.

Pseudocode of the analytical data set construction.

**Table:** 

Algorithm 2. Analytical dataset construction

Input:
• ICD-10 diagnostic variables
• ICD-9-CM procedure variables
• INACBG
• DESCRIPTION
• Length of stay

Process:
1. Retrieve standardized variables from each operational module.
2. All variables are linked using the hospitalization episode identifier.
3. The diagnosis, procedure, reimbursement, and outcome information are combined.
4. One analytical record for each hospitalization episode was generated.
5. Verify the record’s completeness and repository integrity.

Output:
A machine learning-ready analytical repository

### Variable ordering strategy

To maintain a consistent repository structure, diagnosis and procedure variables were organized using a fixed-slot representation. The first diagnosis field (Diagnosis_1) stores the principal ICD-10 diagnosis recorded for each hospitalization episode, followed by secondary diagnoses (Diagnosis_2–Diagnosis_12). Procedure_1 contains the principal ICD-9-CM procedure, followed by the secondary procedures (Procedure_2–Procedure_17). Episodes containing fewer diagnoses or procedures retain structurally missing values, whereas diagnoses or procedures exceeding the predefined repository capacity are excluded from the standardized analytical repository.

### Data quality assurance and consistency verification

Data quality assurance is a critical component of the clinical pathway data engineering framework. Before repository standardization, quality assurance was conducted sequentially to evaluate structural completeness, duplicate hospitalization episodes, diagnosis coding consistency, procedure coding consistency, reimbursement integrity, and logical consistency of hospitalization outcomes. Hospital information systems are primarily designed for operational rather than analytical purposes; therefore, inconsistencies may occur during routine data collection, coding, and reimbursement processes.

This stage aims to identify and correct structural issues that may affect analytical reliability while preserving relevant clinical information.

### Assessment of the missing data

Diagnosis and procedure variables may contain missing values because not every hospitalization episode requires the same number of diagnoses or procedures. Instead of automatically removing incomplete records, CPDEF evaluates missing-value patterns and distinguishes between structurally missing information and unexpected missing data.

Missing structural values occur when additional diagnostic or surgical procedures are not clinically required. Such values are retained because they represent legitimate clinical variability.

### Coding-System verification

Coding verification procedures are applied to ensure consistency between the following:

1) ICD-10 diagnoses.
2) ICD-9-CM procedures.
3) INA–CBG classifications.

Verification includes format checking, completeness assessment, and consistency validation across related variables.

### Outcome verification

To ensure logical consistency, the length-of-stay values are evaluated.

### Validation procedures verify that

1) The LOS values are numerical.
2) LOS values are positive.
3) The LOS values correspond to the number of valid hospitalization episodes.

Pseudocode data quality assurance workflow.

**Table:** 

Algorithm 3. Data quality assurance

Input:
standardized analytical repository

Process:
1. Assess the structural missing values.
2. Identify duplicate hospitalization episodes.
3. Verify the ICD-10 coding format.
4. Verify the ICD-9-CM coding format.
5. Verify the INA-CBG code consistency.
6. Validate the LOS values.
7. A quality-assured analytical repository is generated.

Output:
Quality-assured repository.

### Standardized and documented repository

The final stage of CPDEF focuses on repository standardization and documentation to ensure methodological transparency, reproducibility, and long-term usability. A standardized analytical repository was generated along with supporting documentation describing the repository structure, variable definitions, metadata, validation records, and workflow implementation.

The standardized repository contains 32 analytical variables, including 12 ICD-10 diagnosis variables, 17 ICD-9-CM procedure variables, 2 administrative case-mix variables (INACBG and DESCRIPTION), and 1 hospitalization outcome variable (LOS). Each variable was documented according to data type, coding system, clinical interpretation, and analytical purpose. A complete variable dictionary is provided as Supplementary Material to facilitate repository reuse and independent implementation.

This documentation enables researchers, healthcare analysts, and ML practitioners to consistently reconstruct and reuse the standardized analytical repository.

The resulting repository is suitable for publication within open scientific repositories and supports reproducible healthcare analytics research.

A complete variable dictionary describing all 32 repository variables, including variable names, data types, coding systems, permissible values, and analytical purposes, is provided in the Supplementary Information. This dictionary defines the target repository schema required for the CPDEF methodology’s independent implementation.

### Computational workflow and software environment

The Clinical Pathway Data Engineering Framework was implemented using an open-source computational environment to maximize reproducibility and accessibility.

Data processing was performed using Python-based analytical workflows. The implementation includes data extraction, transformation, quality assurance, validation, and repository construction modules.

The workflow was designed to support execution on standard computing environments without the need for specialized hardware.

### ML readiness validation framework

The final component of CPDEF evaluates whether the standardized analytical repository can be directly incorporated into representative machine learning workflows without requiring additional structural modification. Unlike traditional healthcare data engineering approaches that focus solely on data preparation, CPDEF explicitly incorporates machine learning readiness assessment as the final validation stage. The objective of this assessment is to determine whether the generated repository satisfies the structural requirements for downstream analytical applications and not to compare predictive performance.

The validation framework comprises four dimensions:

• Structural Readiness.
• Coding-System Readiness.
• Reproducibility Readiness.
• Analytical Readiness.

### Structural readiness

Structural readiness evaluates whether the standardized repository satisfies the following minimum structural requirements for machine learning applications:

• Variable completeness
• Data-type consistency
• Record uniqueness
• Repository dimensionality

### Coding-System readiness

Coding-system readiness evaluates whether all standardized clinical coding systems are preserved throughout repository construction, including:

• ICD-10 compatibility.
• ICD-9-CM compatibility.
• INA–CBG compatibility.

### Reproducibility readiness

Reproducibility readiness was assessed using a deterministic workflow execution. Identical input data, software versions, and processing parameters were repeatedly processed to determine whether the framework consistently generated identical repository structures across multiple executions.

Analytical readiness evaluates whether the repository can be transformed into machine learning feature matrices suitable for classification and regression tasks [14].

### Pseudocode ML readiness assessment

**Table:** 

Algorithm 4. Machine learning readiness assessment

Input:
A quality-assured analytical repository

Process:
1. Verify the completeness and structural consistency of the repository.
2. Verify the integrity of ICD-10, ICD-9-CM, and INA-CBG coding.
3. Repeated deterministic workflow runs are executed using identical input data and processing parameters.
4. Machine learning feature matrices are generated.
5. Verify compatibility with representative ML workflows.
Output:
A machine learning-ready analytical repository

### Method validation

Method validation demonstrates that the Clinical Pathway Data Engineering Framework (CPDEF) can consistently transform heterogeneous hospital information system data into a standardized analytical repository suitable for ML applications. Unlike conventional predictive modeling studies, the validation process focuses on structural integrity, coding-system consistency, deterministic workflow execution, and analytical usability rather than predictive performance outcomes.

Validation procedures were conducted using inpatient hospitalization records collected between November 2016 and April 2026. The source dataset contained information on diagnosis and procedure, case-mix reimbursement classifications, and hospitalization outcomes generated through routine healthcare operations.

Structural validation evaluates whether the framework can successfully generate a consistent analytical repository from heterogeneous source data.

The assessment focused on the following:

• Record completeness.
• Variable consistency.
• Dataset dimensionality.
• Repository integrity.

The CPDEF application successfully generated a machine learning-ready repository consisting of 165,391 hospitalization episodes and 32 analytical variables.

The resulting repository preserved all required analytical components and maintained a consistent structure across all hospitalization episodes.

### Validation of clinical coding

The second validation stage evaluates the integrity of the healthcare coding systems within the repository.

Three coding systems were assessed:

1. ICD-10 classifications.
2. ICD-9-CM procedure classifications.
3. INA–CBG reimbursement classifications.

The coding consistency, structural compliance, and integration compatibility were verified by the validation procedures.

The validation results demonstrate that the proposed framework can preserve the integrity of the coding system during dataset construction.

### Reproducibility validation

Reproducibility is a fundamental objective of the CPDEF. In this study, reproducibility was assessed through deterministic workflow execution by repeatedly processing identical source data using the same software environment and processing parameters. This assessment evaluates whether the proposed workflow consistently generates identical analytical repositories under equivalent execution conditions.

The following aspects were evaluated:

• Number of records generated.
• Number of variables generated.
• Repository schema consistency.
• Output stability.

Identical outputs were obtained across all repeated executions.

The identical outputs demonstrate deterministic workflow behavior under identical input data, software versions, and processing parameters. Together with the publicly available implementation, repository schema, variable dictionary, and workflow documentation, these resources support the proposed methodology’s independent reproduction.

### Validation of machine learning readiness

Machine learning readiness validation evaluates whether the repository can be successfully processed using standard machine learning workflows [15].

Unlike predictive modeling studies, this validation does not aim to identify the best-performing algorithm but to verify that the repository supports analytical processing without requiring additional data restructuring.

The repository was evaluated for compatibility with.

• Classification workflows.
• Regression workflows.
• Feature Engineering Pipelines.
• Categorical encoding procedures.

The successful completion of these procedures confirms that the generated repository satisfies the structural requirements for ML applications.

### Validation of the reusability of the repository

The final validation stage evaluates repository reusability.

The generated repository was designed to support multiple analytical applications, including the following:

• Healthcare analytics.
• Clinical pathway analysis.
• Case-mix analytics.
• Length-of-stay prediction.
• Artificial intelligence applications.

The inclusion of diagnostic variables, procedure variables, reimbursement classifications, and hospitalization outcomes within a single analytical structure enables flexible reuse across different healthcare research domains.

### Positioning CPDEF among existing healthcare data engineering frameworks

Standardized healthcare data frameworks, such as OMOP CDM, HL7 FHIR, MIMIC-IV, and DRG-based reimbursement systems, have substantially improved healthcare interoperability, data sharing, and clinical research. However, their primary objectives differ from those of the proposed CPDEF. OMOP CDM focuses on semantic data harmonization, HL7 FHIR supports healthcare information exchange, MIMIC-IV provides a curated research database, and DRG systems primarily support reimbursement. In contrast, CPDEF provides a reproducible data engineering methodology for transforming routine hospital administrative data into standardized machine learning-ready analytical repositories while preserving ICD-10, ICD-9-CM, and INA-CBG coding structures. CPDEF complements existing standards by documenting a transparent repository construction workflow that supports healthcare analytics, clinical pathway analysis, machine learning, and artificial intelligence applications rather than replacing them.

### Limitations

Although the CPDEF provides a reproducible methodology for constructing machine learning-ready case-mix datasets, several limitations should be acknowledged. First, the framework was developed and validated using hospitalization records generated under the INA–CBG reimbursement system. Healthcare institutions operating under alternative reimbursement mechanisms may require modifications to case-mix representation procedures to accommodate local coding and payment structures.

Second, the current implementation focuses on structured healthcare data, including ICD-10 diagnoses, ICD-9-CM procedures, INA–CBG classifications, and LOS information. Other potentially valuable clinical data sources, such as laboratory test results, physiological measurements, medication records, imaging studies, and unstructured clinical notes, were not included in this study. Additional preprocessing procedures may be required when integrating multimodal healthcare data.

Third, the framework was validated using retrospective hospitalization records from a single hospital. Although repeated workflow executions demonstrated deterministic behavior under identical processing conditions, additional validation across multiple healthcare institutions and information system environments would further strengthen the proposed methodology’s generalizability.

Finally, the machine learning readiness validation focused on structural compatibility and analytical usability rather than predictive performance. Therefore, the framework does not prescribe specific ML algorithms and may require task-specific feature engineering depending on the intended analytical application.

## Ethics statements

This retrospective study used administrative hospitalization data collected during routine clinical practice. Before repository construction, all direct patient identifiers were permanently removed through a structured de-identification process. The resulting analytical repository contains no personally identifiable information and was used exclusively for scientific research purposes. The research protocol was reviewed and approved by the **Health Research Ethics Committee (HREC), Universitas Muhammadiyah PKU (UMPKU) Surakarta** under **Ethical Clearance No 231/LPPM/UMPKU/VI/2026 (Protocol ID: 26/06/228)**, issued on **June 08, 2026**. Because this study involved a retrospective analysis of fully de-identified administrative healthcare records, no direct patient contact occurred, and the requirement for individual informed consent was waived in accordance with institutional ethical regulations governing the secondary use of anonymized healthcare data. The publicly released repository contains only anonymized variables and complies with institutional requirements for ethical data sharing and scientific reuse.

## CRediT author statement

Suryanto Nugroho: Conceptualization, Methodology, Software, Data Curation, Formal Analysis, Validation, Visualization, Original Draft Writing, Review, and Editing.

Raden Venantius Hari Ginardi: Conceptualization, Investigation, Validation, Formal Analysis, Review, and Editing.

I Ketut Eddy Purnama: supervision, methodology, validation, writing, review, and editing.

## Declaration of AI-Assisted technologies

The authors exclusively used Trinka AI for language editing and grammatical improvement during manuscript preparation. The authors have reviewed and edited all AI–generated suggestions and have accepted full responsibility for the publication’s content.

## Declaration of Interests

The authors declare that they have no known competing financial interests or personal relationships that could have appeared to influence the work reported in this paper.

## Data Availability

https://doi.org/10.5281/zenodo.20588385.
