# Supplementary material for: Clinical pathway data engineering framework (CPDEF): a reproducible methodology for constructing machine learning-ready case-mix datasets from hospital information systems
Source: MethodsX. 2026 Sep 13;17:104163. doi: 10.1016/j.mex.2026.104163 (PMC13597915; doi:10.1016/j.mex.2026.104163)
Supplement: Supplementary file 1 [file mmc1.docx]

**Supplementary material additional information**

Supplementary Table S1. The standardized 32-variable machine learning-ready repository variable dictionary generated by the Clinical Pathway Data Engineering Framework (CPDEF), including ICD-10 diagnosis variables, ICD-9-CM procedure variables, INA-CBG administrative variables, and the length-of-stay outcome variable.

Table S1. The CPDEF repository variable dictionary shows standardized variable names, data types, clinical descriptions, and representative example values used to construct the machine learning-ready case-mix repository.

| Column name | Data type | Description | Example Record |
| --- | --- | --- | --- |
| Primary_Diagnosis | String | Primary diagnosis using the International Classification of Diseases-10 system | I50.0 |
| Secondary_diag1 | String | Secondary diagnosis using the International Classification of Diseases-10 system | I46.9 |
| Secondary_diag2 | String | Secondary diagnosis using the International Classification of Diseases-10 system | E11.9 |
| Secondary_diag3 | String | Secondary diagnosis using the International Classification of Diseases-10 system | E87.1 |
| Secondary_diag4 | String | Secondary diagnosis using the International Classification of Diseases-10 system | A16.2 |
| Secondary_diag5 | String | Secondary diagnosis using the International Classification of Diseases-10 system |  |
| Secondary_diag6 | String | Secondary diagnosis using the International Classification of Diseases-10 system |  |
| Secondary_diag7 | String | Secondary diagnosis using the International Classification of Diseases-10 system |  |
| Secondary_diag8 | String | Secondary diagnosis using the International Classification of Diseases-10 system |  |
| Secondary_diag9 | String | Secondary diagnosis using the International Classification of Diseases-10 system |  |
| Secondary_diag10 | String | Secondary diagnosis using the International Classification of Diseases-10 system |  |
| Secondary_diag11 | String | Secondary diagnosis using the International Classification of Diseases-10 system |  |
| Primary_Procedure | String | Primary procedure using ICD-9-CM | 96.71 |
| Secondary_proc1 | String | Secondary procedure using ICD-9-CM | 87.49 |
| Secondary_proc2 | String | Secondary procedure using ICD-9-CM | 88.75 |
| Secondary_proc3 | String | Secondary procedure using ICD-9-CM | 87.79 |
| Secondary_proc4 | String | Secondary procedure using ICD-9-CM |  |
| Secondary_proc5 | String | Secondary procedure using ICD-9-CM |  |
| Secondary_proc6 | String | Secondary procedure using ICD-9-CM |  |
| Secondary_proc7 | String | Secondary procedure using ICD-9-CM |  |
| Secondary_proc8 | String | Secondary procedure using ICD-9-CM |  |
| Secondary_proc9 | String | Secondary procedure using ICD-9-CM |  |
| Secondary_proc10 | String | Secondary procedure using ICD-9-CM |  |
| Secondary_proc11 | String | Secondary procedure using ICD-9-CM |  |
| Secondary_proc12 | String | Secondary procedure using ICD-9-CM |  |
| Secondary_proc13 | String | Secondary procedure using ICD-9-CM |  |
| Secondary_proc14 | String | Secondary procedure using ICD-9-CM |  |
| Secondary_proc15 | String | Secondary procedure using ICD-9-CM |  |
| Secondary_proc16 | String | Secondary procedure using ICD-9-CM |  |
| INACBG | String | INACBG case mix code | J-1-20-III |
| DESCRIPTION | String | INACBG description | NON-COMPLEX (SEVERE) RESPIRATORY SYSTEM |
| LOS | Integer | Length of stay (in days) | 3 |
